# Establishing Psychometric Properties of the Modified Barriers Experienced in Providing Healthcare Instrument

**DOI:** 10.3390/healthcare14010102

**Published:** 2026-01-01

**Authors:** Tabarak O. Alomar, Gillian C. Glivar, Eva B. Chung, Kathryn J. Craig, Allie M. Ward, Audrey J. Dingel, B. Kelton Kearsley, Jake R. Goodwin, Allie D. McCurry, Madeline P. Casanova, Alexandra Dluzniewski, Russell T. Baker

**Affiliations:** 1 WWAMI Medical Education Program, School of Health and Medical Professions, University of Idaho, 875 Perimeter Drive MS 4061, Moscow, ID 83844-3020, USA; talomar@uw.edu (T.O.A.); gilliang@uw.edu (G.C.G.); evachung@uw.edu (E.B.C.); craigka@uw.edu (K.J.C.); award26@uw.edu (A.M.W.); dingelau@uw.edu (A.J.D.); bkkears@uw.edu (B.K.K.); jgood1@uw.edu (J.R.G.); addm1@uw.edu (A.D.M.); mcasanova@uidaho.edu (M.P.C.); adluz@uidaho.edu (A.D.); 2 School of Medicine, University of Washington, 1959 NE Pacific St., Seattle, WA 98195-6420, USA; 3 Idaho Office of Underserved and Rural Medical Research, School of Health and Medical Professions, University of Idaho, 875 Perimeter Drive MS 4061, Moscow, ID 83844-4061, USA

**Keywords:** rural healthcare, structural validity, confirmatory factor analysis

## Abstract

**Background:** Rural healthcare providers encounter multifaceted barriers including geographic isolation, resource limitations, and provider shortages that impede optimal patient care delivery. The Barriers Experienced in Providing Healthcare Instrument (BTCPI) was designed to assess provider challenges; however, concerns regarding its psychometric properties necessitated comprehensive validation. The primary purpose of the study was to evaluate the structural validity of the instrument using confirmatory factor analysis with a sample of Idaho healthcare professionals. Because the model failed to meet criteria, the study identified a more parsimonious model that then underwent multi-group invariance testing. **Methods:** A survey consisting of a modified Barriers to Providing Optimal Healthcare instrument and a demographic questionnaire was distributed to Idaho healthcare providers across 22 clinical sites in the state. The structural validity of the modified 41-item, 9-factor instrument was assessed using confirmatory factor analysis (CFA), exploratory structural equation modeling (ESEM), and exploratory factor analysis (EFA). Multi-group invariance testing was also conducted to assess measurement equivalence across provider profession, practice setting (rural vs. urban), and years of experience. **Results:** A total of 373 healthcare providers completed the survey and were used for analysis. The proposed BTCPI model did not meet model fit criteria. An ESEM analysis was conducted and identified a 9-factor, 14-item model. However, due to fit concerns, an exploratory factor analysis was subsequently conducted and identified the 4-factor, 12-item (BPOC-12) that also met invariance criteria across groups. A group mean and variance differences were found between nurses and primary care providers as well as between rural and urban practitioners on several barrier factors. **Conclusions:** The BTCPI did not meet model fit criteria. Subsequent model refinement resulted in the BPOC-12, which had preliminary psychometric validity. Although the refined model offered a more condensed and preliminarily valid psychometric framework, future research should be done to assess this model. Future research should also collect responses from different healthcare professions to enhance its applicability.

## 1. Introduction

Access to healthcare is a fundamental aspect of health and well-being [1]. However, ensuring equitable healthcare access can be challenging when large portions of the United States population, approximately 46.1 million individuals (14% of the total population), live in rural counties [2]. Rural patients face numerous and diverse barriers to healthcare access, which include geographic isolation, transportation, economic instability, provider shortages, discontinuity of care, funding disadvantages, stigma, lack of education about prevention, resource limitations, and ethical challenges [3,4]. Thus, rural patients experience greater health outcome disparities compared to their urban counterparts. For example, rural residents have elevated smoking rates, more sedentary behavior, increased rates of obesity, higher prevalence of suicide and serious mental illness, and overall higher child and young adult mortality [5]. When comparing rural hospitals to their urban counterparts, both hospital readmission rates and post-discharge mortality are higher with mortality difference rates of 0.4% for patients discharged to the community and 2.0% among patients discharged to post-acute care facilities [6]. Additionally, in 2022 alone, approximately 40,000 rural area residents died from a preventable disease due to lack of access to healthcare services, increased rates of poverty, and decreased likelihood of following health promoting practices [7].

As financial pressures force rural hospitals to reduce staff, eliminate departments, or shut down entirely [8], the clinical and administrative burden on remaining providers intensifies, fueling a cycle of burnout, workforce attrition, and diminished access to care [9]. Rural providers are not only grappling with the logistical challenges of delivering care in often under-resourced settings but also face potential systemic, ethical, or emotional barriers that compromise their ability to provide optimal care. Despite the central role providers play in healthcare, their perspectives on these barriers remain underexamined. While much research has been focused on assessing patient-perceived barriers to care [10], far less is known about the challenges perceived by the providers. This information is essential to assist with designing realistic and sustainable solutions. Having a validated instrument to assess the unique challenges faced by providers serving these rural communities is needed to improve optimal patient-centered care and support and strengthen the sustainability of rural health systems.

One potential tool for assessing provider-perceived barriers is the Barriers Experienced in Providing Healthcare Instrument (BTCPI) [3]. The BTCPI was developed using a rigorous mixed-method approach with a sample of healthcare providers in Alaska and New Mexico. Researchers used a grounded theory and narrative analysis to develop preliminary items which were pilot-tested and revised to create an 11-factor, 40-item instrument. The 11 factors identified included Time Constraints (6 items), Resource Limitations (5 items), Confidentiality Limitations (4 items), Training Constraints (6 items), Patient Avoidance of Care (7 items), Service Access (4 items), Outsider Role (3 items), Patient Complexity (3 items), Overlapping Roles (3 items), Language Differences (1 item), and Provider Travel (1 item). Preliminary analyses suggest its viability for use; however, concerns remain regarding its psychometric properties. For example, two factors in the instrument had Cronbach’s alpha values of 0.90 (above the recommended 0.89 criteria), which may indicate item redundancy, while one factor had an alpha below 0.70, indicating low internal consistency [3]. Additionally, two factors comprised only one item each, which does not align with best practices that recommend at least two, preferably three, items per factor [3].

While the instrument has preliminary findings that suggest feasibility to assess barriers to providing optimal care, a complete and robust psychometric analysis of the scale has yet to be conducted [11,12]. Specifically, a confirmatory factor analysis is needed to test the structural validity of the proposed factor model across a broader, more diverse healthcare provider sample [11]. Therefore, the primary purpose of this study was to assess the structural validity of the instrument using classical test theory confirmatory factor analysis (CFA) procedures in a sample of Idaho healthcare professionals. Because model fit did not meet the recommended criteria, the secondary purpose of this study was to identify a more parsimonious model that met fit indices using exploratory structural equation modeling (ESEM), exploratory factor analysis (EFA), and covariance model procedures. The identified refined model then underwent multigroup invariance procedures to assess scale structure between subgroups of interest (i.e., healthcare professional role, years of practice, rurality).

## 2. Materials and Methods

A survey that included a modified Barriers Experienced in Providing Healthcare Instrument (BTCPI) and demographic questionnaire was developed to be disseminated to healthcare providers across the state of Idaho. A convenience sample of healthcare providers were recruited from 22 different clinical sites. Healthcare providers could complete the survey electronically (Qualtrics Inc., Provo, UT, USA) or on a paper form. Prior to completing the survey, participants provided informed consent and were compensated with a $25 Amazon gift card. The University Institutional Review Board determined the study to be exempt.

### 2.1. Instrumentation

#### 2.1.1. Modified Barriers Experienced in Providing Healthcare Instrument (Modified BTCPI)

The original BTCPI [3] was developed to assess how frequently healthcare providers encountered perceived barriers to delivering optimal care in their communities. The original instrument included 40 items, across 11 factors. For our study, 38 of the original items were retained across 9 factors, with the original single item factors excluded. To align with best practices in survey design [13], minor modifications were made to two double-barreled items. These two items were split into separate, more focused questions (i.e., time constraints original item “insufficient time to fully learn about client/patient characteristics, values, or preferences pertaining to their care” was split into “insufficient time to fully learn about client/patient” and “insufficient time to fully learn about client/patient values or preferences pertaining to their care”; training constraints original item “limited time/coverage to have down time or vacation” was split into “limited time/coverage to have breaks during the workday” and “limited time/coverage to have vacation/paid time off”), resulting in a total of 41 items in our modified version. Participants responded to the items using the original 10-point Likert scale, ranging from 0 “never a barrier” to 10 “always a barrier” [3].

#### 2.1.2. Participant Demographic Questionnaire

Participants completed a demographic questionnaire collecting information about their sex, age, ethnicity, profession, specialty field, primary and secondary practice location, current practice status, and years of professional experience.

#### 2.1.3. Data Cleaning and Procedures

Histograms, skewness values, and kurtosis values were used to assess data normality. Z-scores (with a cut-off value of |3.3|) were used to assess univariate outliers and Mahalanobis distance (with a significance level of *p* = 0.01) was used to assess for multivariate outliers. Using the full sample, a confirmatory factor analysis with maximum likelihood estimation was conducted to test model fit on the originally proposed scale [3]. Model fit indices did not meet the recommended criteria [11]; therefore, an (ESEM) analysis was conducted to determine if a more parsimonious model could be identified. Because the ESEM approach did not identify a model that fit all criteria, the dataset was then randomly split into two samples (n1 = 187, n2 = 186) for subsequent analysis. Sample n1 was used to conduct an EFA, with the purpose of identifying a more parsimonious solution. Using sample n2, the EFA solution identified was then tested using a more rigorous covariance model approach [11]. The identified model then underwent CFA multigroup invariance testing using the full sample to complete further necessary psychometric testing of the measurement properties of the proposed scale [11,12].

### 2.2. Data Analysis

#### 2.2.1. Confirmatory Factor Analysis

A CFA with maximum likelihood extraction was conducted on the BTCPI using the Analysis of Moment Structures Version 26 (AMOS, IBM, SPSS, Chicago, IL, USA) software. To evaluate fit of the CFA model, overall goodness of fit indices were evaluated. The model was deemed acceptable if the following contemporary criteria were met: Comparative Fit Index (CFI ≥ 0.95); Tucker–Lewis Index (TLI ≥ 0.95); Root Mean Square Error of Approximation (RMSEA ≤ 0.06); Bollen’s Incremental Fit Index (IFI ≥ 0.95) [10,12]. The likelihood ratio statistic (Chi-square) was evaluated; however, due to its susceptibility to sample size [11,14] the other fit indices were given greater weight. Parameter estimates for factor variances, covariances, indicator errors were also evaluated to assess for localized areas of strain [12].

#### 2.2.2. Exploratory Structural Equation Modeling

Because model fit for the CFA did not meet recommendations (e.g., CFI > 0.95), ESEM was performed to identify a more parsimonious model. ESEM combines the strengths of EFA and CFA because it assesses the relationships among observed variables while simultaneously testing a specified factor structure through estimation of factor loadings, factor correlations, and unique variances, thus providing a more nuanced understanding of the latent factors being measured [15]. ESEM offers a compromise between the more iterative approach through rotations within an EFA and the more restrictive, a priori theory-driven approach within CFA [15]. The same model fit indices used for the CFA were used to evaluate the ESEM model. Item reduction during the ESEM followed recommended practices which balances empirical fit with conceptual clarity. Items were evaluated and removed one at a time using a combination of model fit indices, localized areas of strain, factor interpretability, and theoretical fit [11,12]. Because the ESEM model fit criteria did not meet contemporary standards, the full sample was split into two (n1 = 186; n2 = 187) subsamples to conduct an EFA and covariance model. This approach is considered acceptable when sample size is limited and additional data unavailable [12].

#### 2.2.3. Exploratory Factor Analysis

Sample n1 was used to conduct an EFA and sample n2 was used for a subsequent covariance model using a CFA approach. To find a more parsimonious model, EFA using was conducted on sample n1 with maximum likelihood extraction and direct oblimin rotation. Barlett’s test for sphericity (<0.01) and Kaiser–Meyer Olkin Measure of Sampling Adequacy (≥0.70) were assessed [16]. The criteria used to determine number of factors to retain included: (1) eigenvalues for factors > 1.0; (2) scree plot inflection point examination; and (3) factors accounting for ≥5% of the variance [12,16]. Following extraction, individual items were assessed and removed based on the following guidelines: loading < 0.40; cross-loading ≥ 0.30; low internal consistency; high bivariate correlations with another item; misfit either theoretical or conceptually [12,16,17]. Internal consistency of the factors was assessed using Cronbach’s alpha and omega, with acceptable value criteria set as ≥0.70 and ≤0.89 [16,17].

#### 2.2.4. Validation Analysis of the Modified Scale

Sample n2 was used to assess the identified EFA model fit in a covariance model. The covariance model was evaluated using the same overall goodness-of-fit criteria for the initial CFA [11,12]. Localized areas of strain and Modification indices and localized areas of strain were assessed and if necessary, modifications were made. A correlation analysis between the original modified 9-factor, 41-item BTCPI and the refined version was conducted to assess for criterion validity of the refined BTCPI. A high correlation between scale scores would support criterion validity that the new model was assessing a similar phenomenon as the original model [11].

#### 2.2.5. Multi-Group Invariance Analysis

Using the full sample, multi-group invariance testing was conducted on the identified EFA solution across years of clinical practice (Group 1: <10 years; Group 2: >11 years), town population size (Group 1: rural ≤ 4999 people; Group 2: urban ≥ 50,000 people), and profession (Group 1: PCP = MD/DO, NP, PA; Group 2: Nurse). Invariance testing involved evaluating the configural, metric, and scalar models [11]. The configural model assesses whether the overall model structure remains consistent across subgroups. If the configural model holds, the more restrictive metric model can be assessed, which tests whether factor loadings are equivalent across subgroups. If the metric model holds, the scalar model is then assessed to test if item intercepts are equivalent. The configural model is evaluated using the same cutoff values as those applied in the initial CFA and covariance model. To determine invariance, the metric and scalar model are compared to the configural model values. Invariance is established if the CFI change (CFIdiff) is ≤0.01 [12]. When metric invariance is met, latent variances can be compared across subgroups. If scalar invariance is achieved, latent means can also be compared to detect subgroup differences. However, if CFIdiff exceeds 0.01 for either latent variances or latent means, differences between subgroups cannot be compared as they would be considered distinct [18].

## 3. Results

A total of 457 participants responded to the modified BTCPI; 33 individuals were missing responses to more than 10% of the survey items and were removed from the dataset. Of the remaining 424 participants, eight participants’ responses were identified as univariate outliers (z scores > 3.3), while an additional 43 participant responses were identified as multivariate outliers (Mahalanobis distance > 64.95). The responses from these participants were also removed from the data set, leaving a total of 373 participants for analysis. The included respondents were aged 18–77 (mean age = 42 ± 12 years) with females accounting for 72.9% (n = 272) of the sample. A full breakdown of participant demographics is presented in Table 1.

### 3.1. Confirmatory Factor Analysis

**Figure 1:**
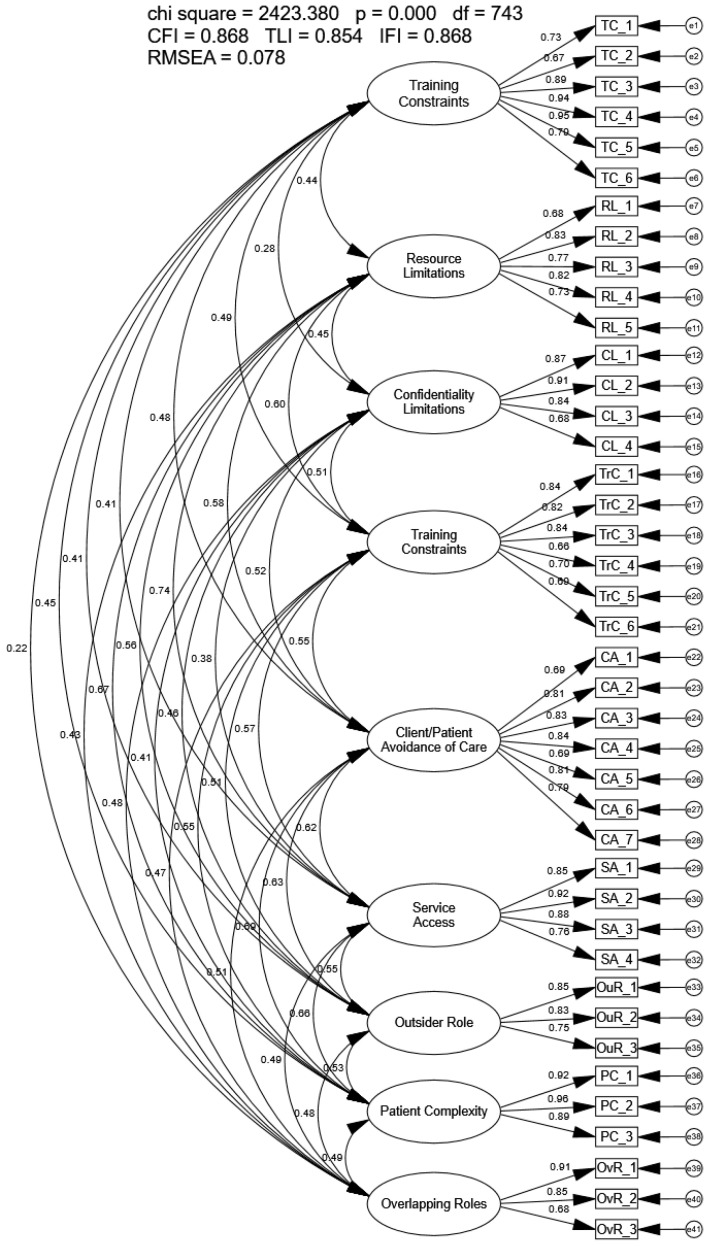
Confirmatory Factor Analysis of the Modified Barriers Experienced in Providing Healthcare Instrument.

The CFA using the full sample of the proposed nine-factor model did not meet recommended goodness-of-fit indices (CFI = 0.868, TLI = 0.854, IFI = 0.868, RMSEA = 0.078; Figure 1). All factor loadings and correlations between latent factors were significant. Factor loadings ranged from 0.66 to 0.95 and correlations between latent factors ranged from 0.22 to 0.74. A review of the modification indices also suggested revisions to the model were necessary. Because model fit indices were not met, extremely high correlations between factors were present, and modification indices suggested revisions, ESEM was conducted using the full sample to identify a more parsimonious model that met recommended fit criteria.

### 3.2. Exploratory Structural Equation Modeling Analysis

**Figure 2:**
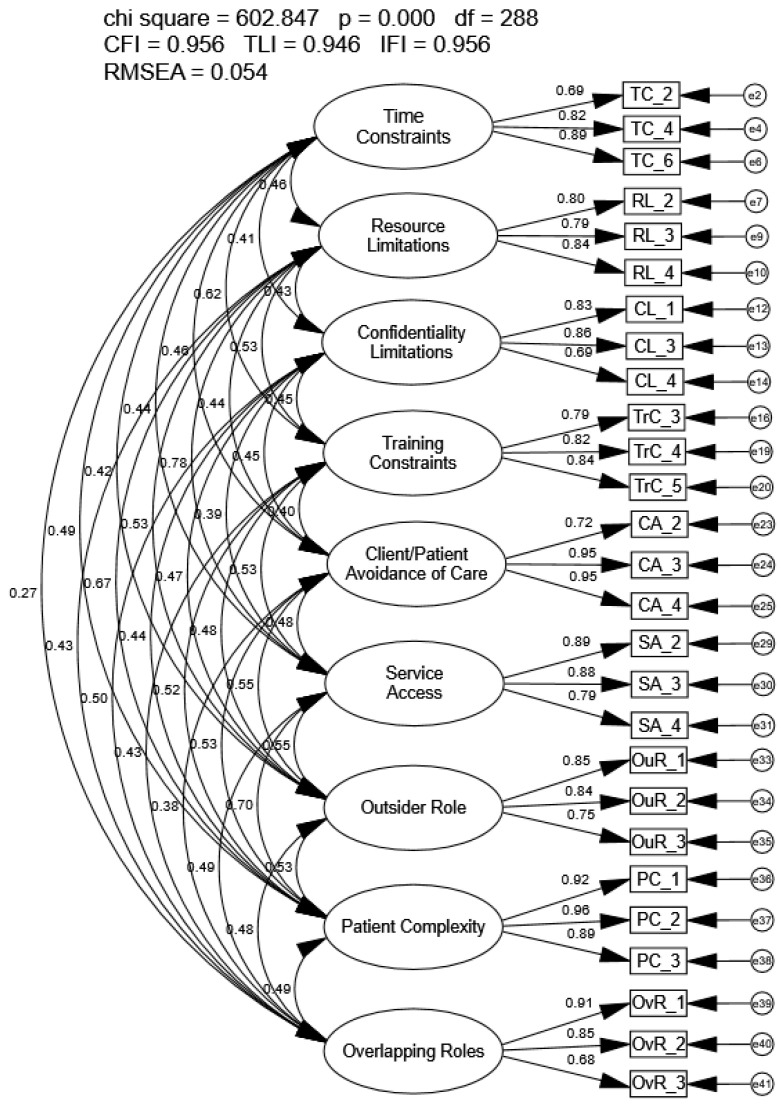
Exploratory Structural Equation Model of the Condensed Modified Barriers Experienced in Providing Healthcare Instrument.

Using modification indices as well as local fit indices, a total of 14 items were removed one at a time. A condensed 9-factor, 27-item model was identified that met most but not all recommended goodness-of-fit indices (CFI = 0.956, TLI = 0.946, IFI = 0.956, RMSEA = 0.054; Figure 2). All loadings were significant and ranged from 0.69 to 0.96. Correlations between latent factors were also significant and ranged from 0.38 to 0.78. While the model had improved model fit compared to the original 41-item model, modification indices suggested revisions to the model were still necessary; further, the high correlations between factors also indicated further revisions to the items. Therefore, EFA was conducted to identify a more parsimonious and psychometrical sound model using sample n1.

### 3.3. Exploratory Factor Analysis

Initial EFA of the modified BTCPI using sample n1 extracted seven factors that accounted for 73.2% of the variance; however, numerous concerns (e.g., high inter-item correlations, item cross-loadings, low item loadings within factors, etc.) were identified in the model. A total of 29 items were removed one at a time due to high inter-item correlations, inflated Cronbach’s alpha levels, and high cross-loadings until a psychometrically sound model was identified. The refined model included 12 items across four factors (Table 2), with all items having sufficient factor loadings (e.g., above 0.50) and all factors having Cronbach’s alpha and omega values within a priori range. Factor 1 included three items from the original “Service Access” factor, factor 2 included three items from the original “Confidentiality Limitations” factor, factor 3 included three items from the original “Time Constraints” factor, and factor four included three items from the original “Outsider Role” factor. The refined model was renamed as the Barriers to Providing Optimal Care instrument (BPOC-12) and was then tested using a covariance model approach with sample n2.

### 3.4. Validation of the Refined Barriers to Providing Optimal Care Model

**Figure 3:**
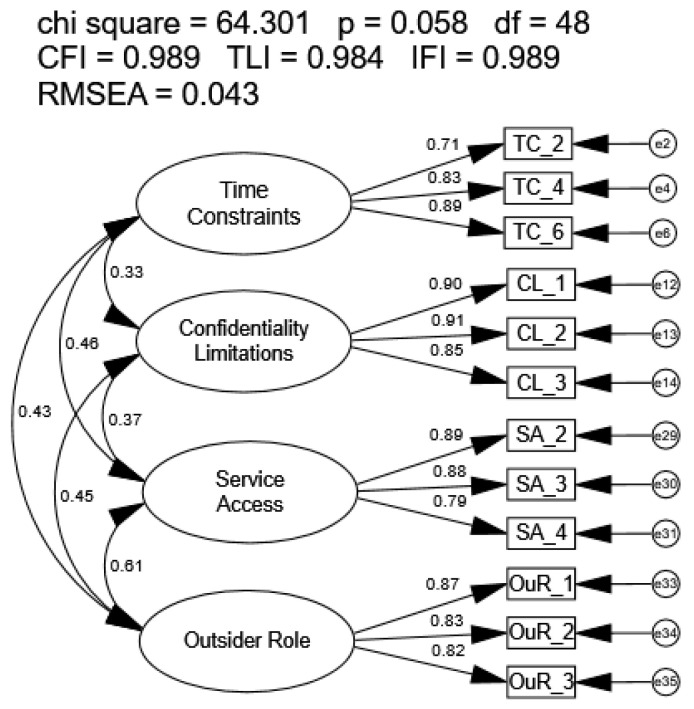
Covariance Model of the Refined Barriers Experienced in Providing Healthcare Instrument.

The covariance model four-factor BPOC-12 using sample n2 met all recommended goodness-of-fit indices (CFI = 0.989, TLI = 0.984, IFI = 0.989, RMSEA = 0.043; Figure 3), with significant loadings that ranged from 0.71 to 0.91. All factor correlations were significant and were within recommended values, with a range from 0.33 to 0.61. A review of the modification indices did not suggest revisions to the model were necessary. The BPOC-12 scores also had a high correlation (r = 0.94; R2 = 0.88) with scores on the 41-item BTCPI scores, indicating the two scales were measuring a similar phenomenon. 

### 3.5. Multi-Group Invariance Testing

#### 3.5.1. Years of Practice

BPOC-12 model was then subjected to multigroup invariance testing across years of practice subgroups (Group 1: ≤10 years; Group 2: ≥11 years), meeting all model fit criteria for the configural, metric, and scalar models (Table 3). Because metric and scalar invariance were met, latent variances and means could be compared across subgroups to determine if meaningful group differences in scores were found between groups. Statistically significant differences for BPOC-12 scores were not found between groups based on years of clinical practice experience (Table 3).

#### 3.5.2. Profession

The BPOC-12 model multigroup invariance testing across profession subgroups (Group 1: Primary Care Provider (PCP) = MD/DO, NP, PA; Group 2: Nurse) met all model fit criteria for the configural, metric, and scalar models (Table 4), which allowed for group differences in BPOC-12 scores to be assessed. Significant group differences were found between the Equal Factor Variances Model and the Equal Latent Means model. Specifically, the MD/DO/PA/NP group exhibited greater variance of latent mean scores in TC and SA, along with a significantly higher latent mean score on the TC factor and a significantly lower latent mean score on the OuR factor compared to the Nurse group. In contrast, the Nurse group demonstrated greater variance in the CL and OuR latent factors. Statistically significant differences were not found in the CL and SA latent mean scores between the profession subgroups.

#### 3.5.3. Rurality

The BPOC-12 model multigroup invariance testing across rurality subgroups (Group 1: rural ≤ 4999 people; Group 2: urban ≥ 50,000 people), met all model fit criteria for the configural, metric, and scalar models (Table 5), which allowed for group differences in BPOC-12 scores to be assessed. Statistically significant group differences in variance and latent mean scores were observed between rurality subgroups. The rural subgroup (i.e., <4999) demonstrated more variance in the CL and OuR latent factors, while the urban subgroup (i.e., >5000) demonstrated more variance in the TC and SA latent factors. The urban subgroup demonstrated a higher mean score on the TC factor compared to the rural subgroup and a lower mean scores on the CL and SA factors compared to the rural subgroup. Statistically significant differences were not found on the OuR latent factor. 

## 4. Discussion

The purpose of the current study was to evaluate the structural validity of the BTCPI using CFA, ESEM, and EFA techniques. The originally proposed BTCPI model did not meet fit indices in our sample; thus, ESEM and EFA analyses were conducted to identify a more parsimonious model. The ESEM analysis identified a 9-factor, 14-item model; however, due to fit concerns, an EFA was subsequently conducted. The EFA identified a 4-factor, 12-item model (i.e., BPOC-12). The refined BPOC-12 model met all fit indices, which warranted invariance testing to ensure invariance across subgroups of interest and to assess if meaningful group differences were present. 

### 4.1. Psychometric Analysis

#### 4.1.1. Confirmatory Factor Analysis

A CFA was conducted on the modified BTCPI model using a sample of Idaho healthcare professionals. The proposed 9-factor, 41-item model did not meet global fit criteria and had localized fit concerns, including high latent variable correlations [11]. The high correlations between the latent factors suggest potential multicollinearity and poor discriminant validity between factors [11,12]. This may be due in part to item wording and the challenge of writing items to measure distinct but related perceived barrier factors. For example, the item ‘lack of appropriate treatment facilities in the community’ (Resource Limitation) may assess a similar phenomenon as the item ‘distances that client/patients needed to travel to receive general care’ (Service Access). A similar issue was noted in a study on healthcare access barriers, where highly correlated items were theoretically related; researchers addressed this by merging or rewording items to enhance parsimony [19]. Because the proposed CFA model did not meet fit indices, further analysis was warranted to identify a more refined or parsimonious model that met global and local fit indices and reduced factor or item redundancy.

#### 4.1.2. Exploratory Structural Equation Modeling Analysis

To identify a more parsimonious model, an ESEM analysis was conducted, which identified a potential 9-factor, 27-item model. The decision to use ESEM was driven by its ability to retain key theoretical factors while improving model efficiency. ESEM is thought to be a balance between the exploratory flexibility of EFA, which iteratively refines factor solutions, and the more restricted, theory-driven approach of CFA, allowing for a more adaptable model without compromising theoretical integrity [15]. However, several limitations persisted through analysis. Contemporary fit indices were not met and correlations between latent variables exceeded recommended values, indicating poor discriminant validity and possible multicollinearity [11,12]. While the ESEM analysis resulted in a model with fit indices closer to recommended values, it did not meet all contemporary criteria. The proposed model successfully retained all nine original theoretical factors while reducing the number of items, making the instrument more efficient and less burdensome for respondents; however, analysis of the model indicated further revisions were necessary to address the statistical and design concerns. Because ESEM still requires specification of a priori structure, these issues suggest that the theoretical model may be misaligned and that a more exploratory approach was appropriate [11,12]. Although the ESEM model provided a more concise and theoretically sound representation of the proposed 9-factor model, its statistical shortcomings suggested additional analysis using EFA was warranted.

#### 4.1.3. Exploratory Factor Analysis and Validation of the Identified Model

To address the concerns identified in the CFA and ESEM model, EFA and covariance modeling were conducted, resulting in a 4-factor, 12-item model (i.e., BPOC-12) that met model fit indices recommendations for the covariance CFA procedures. This refined model retained four of the original nine factors: Time Constraints, Confidentiality Limitations, Service Access, and Outsider Roles. A primary objective in identifying a parsimonious model is to preserve the theoretical integrity of the original framework in measuring the same phenomenon [11,18]. Although the condensed model included only four of the original nine factors and 12 of the original 41 items, the high bivariate correlation (r = 0.94; R^2^ = 0.88) between the scores on the original model and the condensed model indicates that most of the phenomenon measured with the original 41-item scale was accounted for despite only using 12 items. Thus, the new model reduces the item redundancy in the original model, which may have contributed to model fit issues, while also reducing response burden for participants without sacrificing much of the variance in participant responses on the original scale items.

For example, the Time Constraint factor in the condensed model retained two items—‘excessive practice demands on you that reduced your availability to provide care for clients/patients’ and ‘having to work at a pace that was uncomfortable to you’—which were conceptually similar to eliminated items in the Training Constraint factor such as ‘limited time/coverage to allow you to get additional training’ and ‘limited time/coverage to have breaks during the workday.’ This suggests that the refined model effectively captures the proposed key barriers with fewer items, thereby reducing respondent burden and item redundancy. This is important because question length and complexity contribute to respondent fatigue when completing a survey [20], which can lead to less thoughtful responses later in a survey. Furthermore, response fatigue has been shown to affect data quality [20,21], reinforcing the advantages of a more concise instrument. However, there remains a potential trade-off between data quality and the breadth of information collected which necessitates designing instruments that use an effective number of items to measure all factors of interest [21].

Therefore, while the EFA and covariance modeling results support the structural validity of the BPOC-12, its ability to effectively measure the complete phenomenon of potential barriers to providing optimal care in clinical practice requires further investigation. For example, researchers have identified additional factors, such as language barriers, financial barriers, and insurance-related challenges, as significant obstacles to accessing healthcare [19]. Although these findings were based on patient perspectives, similar barriers may impact healthcare professionals’ ability to provide optimal care. Thus, while the current model meets statistical fit criteria and offers a preliminarily theoretically sound framework, it may not effectively capture all the factors associated with barriers to providing optimal care. Further research is needed to ensure it comprehensively captures this phenomenon, and the identification of additional factors and items may be necessary for a comprehensive assessment of barriers to providing optimal care perceived by healthcare professionals. 

### 4.2. Multi-Group Invariance Testing

The BPOC-12 model passed all measurement invariance criteria between groups: years of experience, provider groups, and rurality. Therefore, we were able to examine mean differences in barriers to providing care between these groups.

#### 4.2.1. Years of Experience Groups

Our study did not find significant differences between the years of experience groups (i.e., ≤10 years, ≥11 years), which aligns with the ongoing debate in existing literature. A comprehensive review of 62 studies found that approximately half of the studies (n = 32) reported that physicians in practice for >20 years, compared with those in practice for <10 years, provided lower-quality care, which may be due to reliance on training from early in their careers and reduced adoption of new therapies over time [22]. However, more recent studies indicate that specialized providers with additional training demonstrated higher quality-care, including lower patient fall rates and reduced healthcare-associated infections [23,24]. Additional research has found that better outcomes in pain management were not associated with more years of nursing experience [25]. Another study found that better pain management was associated with nurse’s empathy and whether they employ a more holistic approach and not associated with their education status or years of practice [26]. This study supports the theory that more years of experience does not necessarily equate to more expertise, knowledge, or skills. Previous literature in conjunction with our study suggests the need for further investigation into whether differences in care suspected to be associated with years of experience arise from providers education and training or whether other variables are confounding factors. Despite these mixed findings, our results suggest that years of experience may have less impact in this context, as providers, regardless of experience, appear to face similar systemic and geographic barriers in rural healthcare settings. 

#### 4.2.2. Provider Groups

Significant differences were found between provider groups. The PCP group (i.e., MD/DO/PA/NPs) reported greater variance and higher mean scores on the Time Constraints factor compared to the Nurse group. This aligns with research suggesting that PCPs often experience time pressure during office visits, particularly when gathering history, performing physical exams, and developing treatment plans [27]. Further, time constraints have been linked to increased patient follow-ups, which could also contribute to a persistent cycle of time related barriers [28]. Greater variance within the PCP group could be explained by the diversity in practice settings, provider patient load, or a possible difference in experience. For example, as providers become acclimated to their unique practice environments over time, they can become more efficient and thus time pressure decreases. The PCP group also exhibited greater variance in Service Access compared to the Nurse group, which could be explained by the varying training levels between the provider group and the potential differences in comfort and competence levels in managing more complex patients and diseases. However, no statistically significant differences in Service Access mean scores were observed between these groups, which is not unexpected as both groups work with the same patient populations and encounter similar challenges in this area.

Conversely, the Nurse group reported greater variance and higher mean scores on the Outsider Roles factor compared to the PCP group. The greater mean score in the Nurse group compared to the PCP group may be explained by the provider-patient relationship in these communities. For example, PCPs are often viewed as more permanent fixtures in a community, with over half of some rural populations having the same provider for more than 5 years [29]. While nurses often hold more short-term positions, especially travel nurses who make up 55% of rural and suburban communities and are typically only in a particular community for an average of 13 weeks [30]. The greater variance in responses in the Nurse group may be due to rural nurses’ increased likelihood of commuting and working full-time compared to their urban counterparts, or because many rural nursing positions are staffed by travel nurses, limiting their ability to engage with community leaders and understand local values [30,31]. Additionally, nurses, particularly those in rural areas, may have less formal education than urban nurses [31] and may feel less confident discussing stigmatizing topics with patients. Previous research has highlighted the need for skill-based training to improve nurses’ ability to address sensitive issues such as alcohol use, lifestyle changes, and sexual health [32,33,34].

For the Confidentiality Limitations factor, the nurse group demonstrated greater variance than the PCP group, though no significant difference in mean scores was found. This could be due to the varied nature of nurse-patient interactions, as nurses spend, on average, 37% of their time in direct patient care, and experience a broader range of confidentiality-related concerns [35]. Greater variability may reflect the diversity of work environments and patient relationships that nurses navigate compared to PCPs. Nurses’ varied responses may also relate to the different types of confidentiality challenges nurses manage, including patient concerns about privacy, access to records, or mandatory reporting requirements, which can vary by work environment and across individuals. In addition, travel nurses may be less concerned about confidentiality than some of their coworkers, since they are less likely to have established long-term relationships.

#### 4.2.3. Rurality Groups

Statistically significant differences were found for both variance and mean scores for the Time Constraints factor between rural (population < 4999) and urban (population > 5000) providers. The larger spread of scores in the urban group may be attributed to the greater diversity in practice settings and patient volumes in urban areas, ranging from high-volume clinics to specialized practices [36]. In contrast, rural providers tend to work in more consistent environments with fewer providers and with a smaller patient base. Higher mean scores among urban providers indicate that they perceive greater time constraints than rural providers. Research has found that among 422 family practitioners and general internists, 53.1% reported time pressure during office visits [37]. Additionally, physicians spent 49.2% of their time on electronic health records (EHR) and desk work rather than direct patient care [38].

Rural providers reported higher mean scores on the Service Access factor, with urban providers reporting greater variance. Rural providers scoring higher for Service Access is in line with previous research that consistently shows rural areas face greater healthcare challenges like access to care, lack of primary care providers per capita, and higher rates of poverty associated with limited or no healthcare insurance [39]. A higher variance for urban providers for Service Access could be explained by the urban population being more individualized and largely related to the unique barriers their patient population faces, commonly related to the differences in uninsured patients served [40]. Uninsured patients experience fragmented care due to reduced access to providers and specialists, fear of costs, stigma, and potential language barriers [41]. The patient population served can vary from urban provider to urban provider due to cultural, ethnical, and socioeconomical needs, compared to rural populations which tend to be more homogeneous [42].

The rural group also demonstrated greater variance in Outsider Roles compared to the urban group, though mean scores were not significantly different. Rural areas have strong community values shaped by geographic isolation, agricultural heritage, economic conditions, religion, and behavioral norms [41,43]. The greater variance in how rural providers perceived the Outsider Roles items may depend on provider background and training. For example, research has shown that providers who grew up in a rural community or had experience serving in more rural communities, tend to have greater retention in rural practice than their urban raised and trained counterparts [44]. In addition, urban areas experience more cultural diversity due to migration patterns, which may reduce the pressure to conform to a single set of cultural norms [41]. This broader acceptance of differences could explain why perceptions of outsider roles show less variance among urban providers compared to their rural counterparts. 

For the Confidentiality Limitations factor, urban providers reported significantly lower mean scores, likely due to the greater anonymity afforded by larger populations, where providers are less likely to encounter patients outside of clinical settings. In contrast, rural providers exhibited greater variance, possibly reflecting differences in community structure. In small, tight-knit rural communities, providers may frequently interact with patients outside of clinical settings due to overlapping social roles, whereas those working in more geographically isolated areas or commuting from other cities may experience fewer such encounters [45].

### 4.3. Limitations and Future Research

Although our study provides preliminary evidence of a more parsimonious and psychometrically sound instrument for measuring barriers to providing optimal care and identified meaningful group differences, several limitations should be considered. First, although our sample was diverse, a larger n:q ratio is recommended for robust psychometric testing [11]. Additionally, all analyses were conducted using a single, moderately sized sample, and although the sample was split for the exploratory and confirmatory analyses, separate samples would have been preferable for each stage of validation [12]. Therefore, findings should be considered preliminary until replicated with an independent sample. Future research should validate the scale using a larger sample to enhance generalizability and confirm factor structure. In addition, gathering data from a larger region may yield broader perspectives from healthcare providers in distinct rural and urban landscapes. Second, our sample included a range of healthcare professionals that reflect state workforce distribution, Idaho has approximately 9.03 nurses per 1000 patients compared to 174 physicians per 100,000 patients in Idaho [46,47] with 31% of our sample identifying as nurses. However, our ability to analyze differences across other key provider groups, such as behavioral health professionals and nurse practitioners, was limited due to sample size constraints. Future research should aim to recruit a broader representation of healthcare professionals to capture a more nuanced understanding of profession-specific barriers to care. Additionally, the condensed model may not fully capture the breadth of barriers experienced by providers. For example, language barriers were not assessed in this instrument and could represent a significant barrier to care in communities with more culturally and ethnically diverse populations, as interpretation services may not be readily available. Additional work is needed to identify whether conceptually meaningful domains were omitted and to refine or expand items. Lastly, while our study identified a more parsimonious model through psychometric analysis, a full psychometric evaluation encompassing additional potential barriers is still necessary prior to adoption in research or clinical practice. Further studies should explore external validity, test–retest reliability, longitudinal invariance testing, and the model’s applicability across different healthcare settings to ensure comprehensive measurement of the barriers to providing optimal care. 

## 5. Conclusions

Our study evaluated the structural validity of the BTCPI and identified a more refined instrument for measuring barriers to providing optimal care (i.e., BPOC-12). The original 9-factor, 41-item model did not meet model fit criteria, necessitating further analysis. A condensed, four-factor BPOC-12 was identified that met psychometric and group invariance criteria. Although the refined model demonstrates preliminary structural validity, further research is necessary before widespread adoption. Future studies should explore the instrument’s applicability across diverse healthcare settings, assess additional barriers not captured in the condensed model, and evaluate its clinical relevance. By refining tools to measure provider-reported barriers, healthcare organizations and policymakers can develop targeted interventions to improve access to care, reduce provider burdens, and promote health, particularly for rural communities.

## Figures and Tables

**Table 1:** Participant Demographic Information.

Demographic Characteristics	Number of Participants	Percentage of Participants (%)
Profession		
Nurse (e.g., RN)	115	30.8
Physician (i.e., MD/DO)	62	16.6
Physician Assistant (i.e., PA)	22	5.9
Nurse Practitioner (i.e., NP)	13	3.5
Social Worker (e.g., LCSW)	7	1.9
Pharmacist (i.e., PharmD)	6	1.6
Other *	134	35.9
Unknown	8	2.1
Years of Clinical Practice		
≤10 years	172	46.1
≥11 years	180	48.3
Unknown	21	5.6
Sex		
Female	272	72.9
Male	91	24.4
Prefer not to answer	2	0.5
Unknown	8	2.1
Race or Ethnicity		
White	311	83.4
Hispanic or Latino or Spanish Origin	33	8.8
American Indian or Alaska Native	7	1.9
Black or African American	3	0.8
Asian	5	1.3
Native Hawaiian or Other Pacific Islander	3	0.8
Other	5	1.3
Prefer not to answer	12	3.2

* (e.g., Respiratory Therapist, Medical Assistant, Occupational Therapist, Certified Nurse Assistant, etc.).

**Table 2:** Exploratory Factor Analysis of the Refined Barriers Experienced in Providing Healthcare Instrument.

Item	1	2	3	4
SA_2	0.913			
SA_3	0.905			
SA_4	0.702			
CL_2		−0.959		
CL_1		−0.827		
CL_3		−0.762		
TC_6			0.841	
TC_4			0.817	
TC_2			0.693	
OuR_3				−0.942
OuR_1				−0.725
OuR_2				−0.612
Eigenvalue	4.873	1.679	1.554	1.318
Cronbach’s alpha	0.884	0.891	0.834	0.816
Omega	0.886	0.892	0.845	0.826

**Table 3:** Multi-group Invariance Testing Between Years of Practice Groups.

	χ^2^	df	χ^2^_diff_ (df_diff_)	CFI	CFI_diff_	TLI	IFI	RMSEA
≤10 (n = 172)	86.79	48	—	0.972	—	0.961	0.972	0.069
≥11 (n = 180)	50.10	48	—	0.998	—	0.998	0.998	0.016
Configural Model (equal form)	136.90	96	—	0.984	—	0.978	0.984	0.035
Metric Model (equal loadings)	140.00	104	3.10 (8)	0.986	0.002	0.982	0.986	0.031
Equal Factor Variances Model	175.23	111	38.33 (15)	0.975	0.009	0.970	0.975	0.041
Scalar Model (equal indicator intercepts)	149.45	112	12.55 (16)	0.985	0.001	0.983	0.985	0.031
Equal Latent Means Model	166.51	116	29.61 (20)	0.980	0.004	0.977	0.980	0.035

χ^2^ = chi square; df = degrees of freedom; χ^2^_diff_ = chi square change; df_diff_ = degrees of freedom change; CFI = Comparative Fit Index; CFI_diff_ = Comparative Fit Index change; TLI = Tucker–Lewis Index; IFI = Bollen’s Incremental Fit Index; RMSEA = Root Mean Square Error of Approximation; — Indicates the value is not calculated at this step.

**Table 4:** Multi-group Invariance Testing Between Profession Groups.

	χ^2^	df	χ^2^_diff_ (df_diff_)	CFI	CFI_diff_	TLI	IFI	RMSEA
MD/DO/NP/PA (n = 97)	57.99	48	—	0.984	—	0.977	0.984	0.047
Nurse (n = 115)	57.02	48	—	0.982	—	0.982	0.987	0.041
Configural Model (equal form)	115.02	96	—	0.985	—	0.980	0.986	0.031
Metric Model (equal loadings)	125.98	104	10.96 (8)	0.983	0.002	0.978	0.983	0.032
Equal Factor Variances Model	147.53	111	**32.51 (15)**	0.971	**0** **.014**	0.966	0.972	0.040
Scalar Model (equal indicator intercepts)	141.51	112	26.49 (16)	0.977	0.008	0.973	0.977	0.035
Equal Latent Means Model	168.73	116	**53.71 (20)**	0.959	**0** **.026**	0.953	0.959	0.047

χ^2^ = chi square; df = degrees of freedom; χ^2^_diff_ = chi square change; df_diff_ = degrees of freedom change; CFI = Comparative Fit Index; CFI_diff_ = Comparative Fit Index change; TLI = Tucker–Lewis Index; IFI = Bollen’s Incremental Fit Index; RMSEA = Root Mean Square Error of Approximation; —Indicates the value is not calculated at this step; Bolded CFI_diff_ indicates criterion has been exceeded; Bolded χ^2^ difference indicates criterion has been exceeded.

**Table 5:** Multi-group Invariance Testing Between Rurality Groups.

	χ^2^	df	χ^2^_diff_ (df_diff_)	CFI	CFI_diff_	TLI	IFI	RMSEA
<4999 (n = 97)	65.41	48	—	0.991	—	0.988	0.991	0.036
>5000 (n = 115)	57.03	48	—	0.985	—	0.979	0.985	0.048
Configural Model (equal form)	122.69	96	—	0.990	—	0.986	0.990	0.028
Metric Model (equal loadings)	131.21	104	8.52 (8)	0.989	0.001	0.987	0.990	0.027
Equal Factor Variances Model	178.17	111	**55.48 (15)**	0.974	**0** **.016**	0.974	0.974	0.041
Scalar Model (equal indicator intercepts)	142.72	112	20.03 (16)	0.988	0.002	0.986	0.988	0.027
Equal Latent Means Model	205.02	116	**82.33 (20)**	0.966	**0** **.024**	0.961	0.966	0.046

χ^2^ = chi square; df = degrees of freedom; χ^2^_diff_ = chi square change; df_diff_ = degrees of freedom change; CFI = Comparative Fit Index; CFI_diff_ = Comparative Fit Index change; TLI = Tucker–Lewis Index; IFI = Bollen’s Incremental Fit Index; RMSEA = Root Mean Square Error of Approximation; —Indicates the value is not calculated at this step; Bolded CFI_diff_ indicates criterion has been exceeded; Bolded χ^2^ difference indicates criterion has been exceeded.

## Data Availability

The dataset used and analyzed in this study may be available from the corresponding author with permission from the University of Idaho upon reasonable request. The data are not publicly available due to study protocol restrictions.
